# Photoprotective energy dissipation is greater in the lower, not the upper, regions of a rice canopy: a 3D analysis

**DOI:** 10.1093/jxb/eraa411

**Published:** 2020-09-09

**Authors:** Chuan Ching Foo, Alexandra J Burgess, Renata Retkute, Pracha Tree-Intong, Alexander V Ruban, Erik H Murchie

**Affiliations:** 1 Division of Plant and Crop Sciences, School of Biosciences, University of Nottingham, Sutton Bonington, UK; 2 Department of Plant Sciences, University of Cambridge, Cambridge, UK; 3 School of Biological and Chemical Sciences, Queen Mary University of London, London, UK; 4 University of Essex, UK

**Keywords:** Canopy, chlorophyll fluorescence, gas exchange, photoinactivation, photosynthesis, productivity, protective non-photochemical quenching (pNPQ), PsbS, rice (*Oryza sativa*)

## Abstract

High light intensities raise photosynthetic and plant growth rates but can cause damage to the photosynthetic machinery. The likelihood and severity of deleterious effects are minimised by a set of photoprotective mechanisms, one key process being the controlled dissipation of energy from chlorophyll within PSII known as non-photochemical quenching (NPQ). Although ubiquitous, the role of NPQ in plant productivity is important because it momentarily reduces the quantum efficiency of photosynthesis. Rice plants overexpressing and deficient in the gene encoding a central regulator of NPQ, the protein PsbS, were used to assess the effect of protective effectiveness of NPQ (pNPQ) at the canopy scale. Using a combination of three-dimensional reconstruction, modelling, chlorophyll fluorescence, and gas exchange, the influence of altered NPQ capacity on the distribution of pNPQ was explored. A higher phototolerance in the lower layers of a canopy was found, regardless of genotype, suggesting a mechanism for increased protection for leaves that experience relatively low light intensities interspersed with brief periods of high light. Relative to wild-type plants, *psbS* overexpressors have a reduced risk of photoinactivation and early growth advantage, demonstrating that manipulating photoprotective mechanisms can impact both subcellular mechanisms and whole-canopy function.

## Teaser

The distribution of protective non-photochemical quenching (NPQ) in rice indicates higher protective capacity in lower canopy layers, regardless of PsbS level, NPQ capacity, or canopy structure.

## Introduction

Photosynthetic efficiency is a limitation to achieving the increases in crop productivity needed to meet the demands of an expanding population. However, we lack an understanding of how canopy structure and internal biochemistry combine to determine the absorption and utilization of light, particularly within the field setting. The within-canopy light environment is highly dynamic, with up to a 50-fold difference in light intensity reaching leaves at the top of the canopy compared with those at the bottom (Niinemets and Keenan, 2012). This is further confounded by changes in canopy architecture, such as leaf angle or area, and shading effects brought about by overlapping foliage, solar movement, cloud cover, and displacement of leaf material (Burgess *et al*., 2016, 2017*b*; Townsend *et al.*, 2018a). The complex and fluctuating light environment poses a problem for the photosynthetic machinery; with the need to maximize the efficient harvesting and utilization of light energy whilst minimizing any deleterious effects associated with exposure to high light.

A number of mechanisms are employed by plants in order to limit damage to the photosynthetic machinery caused by high light; however, through their action, they momentarily reduce the quantum use efficiency of photosynthesis, thus themselves limiting potential productivity. Here, we use the term photoinactivation to describe the light-induced inactivation and therefore functional closure of reaction centres (RCs), including damage, which leads to a decrease in the yield of PSII (ΦPSII; Sonoike, 1996; Takahashi and Badger, 2011; Matloobi, 2012; Murata *et al.*, 2012; Ruban, 2016). The susceptibility of a leaf to photoinactivation depends upon multiple factors including life history (e.g. growing conditions), genetic adaption, and physiological status (Aro *et al.*, 1993; Murchie and Niyogi, 2011; Demmig-Adams *et al.*, 2012). The process employed by plants to relieve excitation pressure on the photosynthetic membrane is non-photochemical quenching (NPQ) of chlorophyll fluorescence, in which excess energy is dissipated harmlessly as heat (Horton and Ruban, 1992; Jahns and Holzwarth, 2012; Ruban, 2016; Murchie and Ruban, 2020). The fastest component of NPQ is qE, or energy-dependent quenching, and is triggered by the generation of a pH gradient across the thylakoid membrane (Krause, 1974; Horton *et al.*, 2005; Zulfugarov *et al.*, 2007). qE is also known to be modulated by the carotenoid zeaxanthin and the protein PSII subunit S (PsbS), which act as allosteric regulators to alter the structure of the membrane and antenna conformation in order to enhance the affinity for protons, thus facilitating qE formation and relaxation (Niyogi *et al.*, 2005; Johnson and Ruban, 2010, 2011; Kereïche *et al.*, 2010; Murchie and Niyogi, 2011; Goral *et al.*, 2012; Harbinson, 2012; Roach and Krieger-Liszkay, 2012; Ruban, 2012, 2016, 2017; Zaks *et al.*, 2012; Sacharz *et al.*, 2017).

Whilst qE formation is rapid (within seconds), the decay is not instantaneous, thus leading to a lag time between changes in light intensity and energy dissipation. Model simulations indicate that this lag time can reduce CO_2_ fixation by between 7.5% and 30%, thus representing a potential route of increasing photosynthetic efficiency (Werner *et al.*, 2001; Zhu *et al.*, 2004). However, it is not known how much NPQ can be considered to be protective; that is, maintain maximal photosynthesis without functional closure of RCs. Overprotection will lead to a reduction in quantum yield, but underprotection might jeopardize photosynthetic efficiency further as repair of RCs is considered to be costly (Raven, 1989). In recent years, there has been increasing interest in manipulating NPQ to improve productivity (Murchie and Niyogi, 2011; Hubbart *et al*., 2012, 2018; Kromdijk *et al.*, 2016; Głowacka *et al.*, 2018). Kromdijk *et al.* (2016) achieved a 15% increase in biomass of field-grown tobacco plants through the overexpression of three genes involved in NPQ. This increase was attributed to increased speed of formation and relaxation of qE. Similar improvements can also be achieved by manipulation of single genes involved in the mechanism. Studies by Hubbart *et al*. (2012, 2018) indicated that overexpression of *psbS* alone can enhance qE and biomass production in rice through increased canopy radiation use efficiency during fluctuating light. However, the influence of altered NPQ components on the distribution of photoprotective capacity throughout the canopy, particularly in relation to structural traits, has not been explored.

NPQ is not an on/off switch but rather is adjusted quantitatively, diverting energy away or towards PSII and, as such, it can exert some regulation over the PSII redox state. It is often observed in the absence of photoinactivation; hence it may operate but have no influence over the prevention of photoinactivation of PSII (Ruban and Belgio, 2014; Ruban, 2017). Given that a persistent NPQ will limit productivity in fluctuating light where photoinhibition is not a risk (Kromdijk *et al.*, 2016), an important question arises: how much NPQ is actually required to prevent the onset of photoinactivation (Ruban, 2016)? A relatively rapid, non-destructive protocol was developed for the measurement of NPQ that quantifies the amount of ‘protective’ NPQ; that is, the amount required to prevent photoinactivation (termed pNPQ; Ruban and Murchie, 2012). The protocol requires no dark adaptation and entails a gradually increasing actinic light (AL) routine to track the relationship between ΦPSII, NPQ, and qP (Murchie and Lawson, 2013) (the quantum coefficient of photochemical quenching) measured in the dark following light exposure (termed qP_d_). Assuming that there is no photoinactivation, qP_d_ should be 1. This parameter can be used to define pNPQ—the NPQ and corresponding AL intensity after which all RCs remain active (i.e. open). This method provides a number of advantages over previous methods and allows a quantitative approach to define the relationship between photoinactivation, NPQ, and the contribution to the decline in ΦPSII. qP_d_ provides a prompt marker of both initial and long-term photoinactivation as it reflects the true state of RCs, enabling the tracking of the early signs of their loss of activity. This method has been successfully used for detection of the early signs of photoinactivation (Ruban and Murchie, 2012; Ruban and Belgio, 2014; for reviews, see Ruban, 2016, 2017). The pNPQ protocol has been used extensively within *Arabidopsis thaliana* to study the contribution of photoprotection versus photoinhibition (Townsend *et al.*, 2018b); the contribution of PSI fluorescence to NPQ (Giovagnetti and Ruban, 2015); and the role of carotenoids and components of NPQ in light tolerance (Ware *et al*., 2015a, b, 2016). It is therefore an ideal method to study the distribution of pNPQ within crop canopies.

Many crops are cultivated as complex, monocropped canopies and so variation in canopy architectural traits poses difficulties in scaling up cellular levels processes to infer canopy function. Recent advances in using realistic three-dimensional (3D) reconstructions and modelling approaches have provided a means to account for canopy traits when assessing cellular level processes (Burgess *et al*., 2015, 2017*b*; Gibbs *et al.*, 2019). When this is coupled with the pNPQ technique, the light tolerance at different canopy positions according to realistic structure can be assessed. This work would divulge information on the ‘cost’ of photoprotection in terms of the productivity of rice canopies. Here, we have applied these methods within rice with the aim to (i) evaluate the distribution of pNPQ according to canopy structure and the in-canopy light environment; and (ii) evaluate the role of *psbS* in pNPQ capacity and distribution.

## Materials and methods

### Plant material, experimental design, and physiological measurements

*Oryza sativa* L. ‘Kaybonnet’ rice wild-type (WT), *psbS*-overexpressing (OE99), and *PsbS-*deficient RNAi lines (RNAi134; *psbS* genomic sequence: Os01g64960) provided by Syngenta (Research Triangle Park, NC, USA) were used in this study. The overexpression of the transgene in these lines was confirmed in a study carried out in the same facility (Hubbart *et al.*, 2018). Plants were sown in the FutureCrop Glasshouse facilities, University of Nottingham, Sutton Bonington Campus (52°49'59''N, 1°14'50''W), UK on 25 April 2017. It is a south-facing glasshouse constructed to contain two 5 m (area) by 5 m by 1.25 m (depth) tanks at ground level (CambridgeHOK, Brough, UK); a single tank was used for this experiment. The tank was filled with sandy loam soil which was extracted from local fields and sieved through a fine mesh. Seeds of the WT (Kaybonnet), OE (OE99), and RNAi (RNAi134) lines were sown into modular trays in a compost mix consisting of 50% John Innes Number 1 and 50% Levington M3. On 10 May 2017 (15 d after sowing), the seedlings were transplanted into a prepared soil bed in a randomized block design with four replicates. Each plot consisted of 8×7 plants, spaced 12 cm apart. A photoperiod of 12 h (07.00 h to 19.00 h) was maintained using blackout blinds. Additional lighting was supplied using sodium (Son T-Agro, Philips) lamps located 3 m above ground height whenever the photosynthetically active radiation (PAR) fell below 200 μmol m^−2^ s^−1^. For small plants, this provides ~100 μmol m^−2^ s^−1^ at plant height, and for the largest plants this provides ~150–200 μmol m^−2^ s^−1^. An automatic drip irrigation was applied for 30 min, twice daily. A temperature of 30±3 °C and relative humidityof 50–60% were maintained throughout.

Plant height, tiller number, and fractional interception were measured weekly. Fractional interception was measured using an AccuPAR LP-80 ceptometer (Decagon Devices, Washington, USA). Measurements were taken at midday after manually switching off the supplementary lighting in the glasshouse. Five measurements were taken diagonally across each plot at each layer. Three plants per plot were harvested after the end of the second (5 July 2017) and third round (24 July 2017) of measurements for dry weight records. Samples were bagged and oven-dried at 70 °C for 48 h until a stable weight was observed.

### Chlorophyll fluorescence and gas exchange measurements

The rice canopies were studied as a two-layered canopy (referred to as top and bottom, respectively), using the height at the centre of each plot as a reference point. Chlorophyll fluorescence and gas exchange measurements were collected at three different growth stages (GSs) hereby known as GS1, GS2, and GS3, corresponding to 35, 50, and 75 d after transplanting (DAT), respectively, on attached leaves. These stages span from tillering to late stem elongation phases (knowledgebank.irri.org). At each stage, a mini PAM fluorometer (Heinz Walz GmbH, Effeltrich, Germany) was used to measure dark-adapted *F*_v_/*F*_m_ (maximum photochemical quantum yield of PSII) at midday. Five leaves per layer of each plot were dark adapted using clips for 25 min. No significant difference was found between any line at any growth stage, with values of ~0.83 indicating maximum functioning of PSII. ANOVA and post-hoc Tukey’s multiple comparisons test was carried out using the statistical package, Genstat (19th Edition) for Windows (VSN International Ltd, Hemel Hempstead, UK). Data were checked to see they had met the assumption of constant variance and normal distribution of residuals.

All the following gas exchange and fluorescence measurements were conducted in the hours around midday for consistency. Measured leaves were randomly selected from each genotype and layer to prevent bias resulting from measurement time. NPQ induction and gas exchange measurements were carried out using a LiCOR 6400XT infra-red gas exchange analyser on attached leaves (LiCOR Biosciences, Lincoln, NE, USA). The block temperature was kept at 30 °C, humidity was set to ambient, and carbon dioxide concentration was maintained at 400 ppm at a flow rate of 500 ml min^–1^. All the light was provided by a combination of in-built red and blue light-emitting diodes (LEDs; set to 10% blue). The youngest fully extended leaf within the designated half of the canopy was chosen and dark adapted with aluminium foil for an hour prior to measurements. An hour-long automated NPQ induction protocol was developed, consisting of an initial log of dark-adapted chlorophyll fluorescence parameters, followed by 15 min of induction at 1500 μmol m^–2^ s^–1^ and 5 min of relaxation by reducing light to 200 μmol m^–2^ s^–1^, with measurements taken every minute throughout. Immediately after the induction protocol, a light response curve was taken. Illumination occurred over a series of 11 PAR values between 0 and 2000 µmol m^–2^ s^–1^ (low to high), with a minimum of 2 min and a maximum of 3 min at each light intensity to enable signal stability and matching between sample and reference chambers performed at every measurement.

#### Protective NPQ

**Fig. 1.**
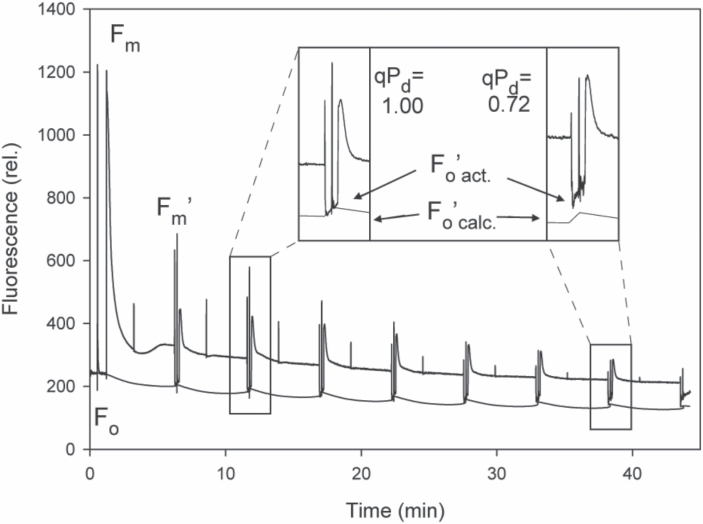
Example scheme of induction of chlorophyll fluorescence with an eight step actinic light (AL) routine made on a Junior-PAM (Heinz Walz). For a detailed explanation of the routine development, see Ruban and Belgio (2014). Inset: the gradually increasing AL routine induces photoinactivation which can be readily observed as a divergence between *F*_o_'_act_ and *F*_o_'_calc_ and a resulting decrease in the qP_d_ parameter.

The theory behind the pNPQ protocol is given in Ruban and Murchie (2012) with more details given in Ruban and Belgio (2014). pNPQ measurements were made using a Junior-PAM fluorimeter (Walz, Effeltrich, Germany) and magnetic leaf clip. Leaves were dark adapted for an hour prior to measurement. The pNPQ procedure was run as a pre-programmed batch file where the scheme was (SP)–(AL on)–(120 s)–(SP)–(180 s)–(SP)–(AL off/FR on)–(7 s)–(SP)–(5 s)-(AL on/FR off)–repeat; where AL is the actinic light, SP is the saturating pulse, and FR is far-red light (Fig. 1). The light intensity emitted from the fibre optic was calibrated using a mini quantum sensor (MQS-B/A; Walz) attached to the universal light meter (ULM-500; Walz). The assessment procedure used AL intensities of 0, 180, 380, 570, 840, 1250, 1690, 2300, and 3000 μmol m^–2^ s^–1^. Intensities of 83.3% and 66.7% for each light step (correlating to a maximum of 2500 μmol m^–2^ s^–1^ and 2000 μmol m^–2^ s^–1^, respectively) were also used for a greater representation of leaf variation by manually adjusting the AL setting in the Walz software. In order to account for the natural variations in qP_d_ values between leaves, a qP_d_ of 0.98 was selected as a mark of photoinactivation, meaning that 2% of RCIIs are functionally closed and the closure is relatively proportional to the decrease of qP_d_. Such values can be used to calculate light tolerance curves, the percentage of the leaves that show a qP_d_ value >0.98, and thus are photoinactivated at each light intensity, as:

$$\begin{array}{l} 100\times \frac{N_{qPd}<0.98}{N_{total}} \end{array}$$ (1)

Ten repetitions were made per light intensity set (i.e. to a maximum of 3000, 2500, and 2000 μmol m^–2^ s^–1^), per canopy layer, and per line for each growth stage in order to build light tolerance curves; in other words, 30 sets of measurements were used to build each tolerance curve, correlating to 10 replicates for 24 light intensities. This results in two canopy light tolerance curves; for the upper and lower canopy, respectively. A regression analysis was performed in Mathematica 10.0 (Wolfram Research Inc., Illinois, USA) to determine the relationship between the percentage of photoinactivated leaves (*p*) and light intensity (*L*), using a sigmoidal Hill function with five parameters:

$$\begin{array}{l} f\left(L\right)=a-\frac{a-b}{{\left\{1+exp\left[c\left(L-d\right)\right]\right\}}^{e}} \end{array}$$ (2)

Five parameters were chosen to accommodate asymmetry and allow greater flexibility when fitting to data. Parameters were fitted to experimental data using a least squared method using the Mathematica function FindFit. Photolerance can thus be separated into 10% bins, with corresponding light intensity boundaries given by the inverse function, which provides an estimate for the light intensity which gives a set percentage of photoinactivated leaves:

$$\begin{array}{l} L\left(\;p\right)=\frac{c\;\;\;d-log\left(\frac{1}{{\left(\frac{a-b}{a-p}\right)}^{1/e}-1}\right)}{c} \end{array}$$ (3)

### Canopy reconstruction and modelling

3D analysis of plants was made according to the protocol of Pound *et al.* (2014) with further details given in Burgess *et al.* (2015). One rice plant per plot (i.e. four per line) was selected at each of the growth stages and carefully removed for imaging. Water was supplied to the roots to prevent wilting. At least 40 images per plant were taken and reconstructions made as described in Burgess *et al.* (2015). Reconstructed canopies were formed by duplicating and randomly rotating the three best reconstructed plants in a 3×3 grid, with 12 cm between plants, within and between rows in accordance with the planting pattern. Each reconstructed canopy is formed of a set of triangles.

Total light per unit leaf area was predicted using a forward ray-tracing algorithm implemented in fastTracer (version 3; PICB, Shanghai, China; Song *et al.*, 2013). Latitude was set at 53 (for Sutton Bonington, UK), atmospheric transmittance 0.5, light reflectance 7.5%, light transmittance 7.5%, and days 164, 180, and 205 (13 June, 29 June, and 24 July). FastTracer3 calculates light as direct, diffused, and transmitted components separately; these were combined together to give a single irradiance level for all canopy positions. The diurnal course of light intensities over a whole canopy was recorded in 1 min intervals. The ray-tracing boundaries were positioned within the plants on the outside so as to reduce boundary effects.

All modelling was carried out in Mathematica (Wolfram Research Inc.). Cumulative leaf area index (cLAI; leaf area per unit ground area as a function of depth) was calculated from each of the canopy reconstructions. For each depth (*d*; distance from the highest point of the canopy), all triangles with centres lying above *d* were found (Equation 4).

$$\begin{array}{l} d_{i}=max_{j=1,2,3;1\leq i\leq n}z_{i}^{\;j}-\left(z_{i}^{1}+z_{i}^{2}+z_{i}^{3}\right)/3 \end{array}$$ (4)

The sum of the areas of these triangles was calculated and divided by the ground area. The cLAI as a function of depth through the canopy was calculated using Equation 5.

$$\begin{array}{l} cLAI\left(d\right)=\frac{\overset{n}{\underset{i=1}{\sum }}I\left(d_{i}\leq d\right)S_{i}}{\left(\max_{1\leq i\leq n}x_{i}-\min_{1\leq i\leq n}x_{i}\right)\left(\max_{1\leq i\leq n}y_{i}-\min_{1\leq i\leq n}y_{i}\right)} \end{array}$$ (5)

where *I*(*A*)=1 if condition *A* is satisfied and *S*_*i*_ is the area of a triangle *i*.

In order to calculate fractional interception within a canopy as a function of depth (known as cumulative fractional interception; cF) at time *t*, all triangles lying above depth *d* were identified (Equation 6). Their contribution to intercepted light was then calculated by multiplying photosynthetic photon flux density (PPFD) received per unit surface area (ray-tracing output) by the area of the triangle. The light intercepted was summed for all triangles above the set *d*, and divided by light intercepted by ground area according to Equation 6.

$$\begin{array}{l} cF(d,t)=\frac{\overset{n}{\underset{i=1}{\sum }}I\left(d_{i}\leq d\right)\;\;\;S_{i}L_{i}\left(t\right)}{L_{0}\left(t\right)\times ground\;\;\;area} \end{array}$$ (6)

where *L*_0_(*t*) is light received on a horizontal surface with a ground area $\left(\max_{1\leq i\leq n}x_{i}-\min_{1\leq i\leq n}x_{i}\right)\left(\max_{1\leq i\leq n}y_{i}-\min_{1\leq i\leq n}y_{i}\right)$, and *L*_*i*_(*t*) is light intercepted by a triangle *i*.

Profiles of the pNPQ capacity of canopies can be constructed by separating the percentage of photoinactivated leaf area into bins; the corresponding limits of light intensity *L*_*i*_=*L*(*p*_*i*_) can be found in Equation 3. Based on the light intensities computed from the ray tracer, the fraction of a surface having light intensity within each assigned bin at each time point, *t* can be calculated.

## Results

### Canopy architecture and development

Three different growth stages were selected to study the role of pNPQ throughout rice canopy development. There were no significant differences in fractional interception between the OE, RNAi, and WT lines at any growth stage and so measurements were taken at the same time (see Supplementary Fig. S1 at *JXB* online). The first growth stage (GS1) was prior to canopy closure (*f*≥0.6), followed by the second growth stage (GS2) during the canopy closure stage (*f*≥0.8), and the last growth stage (GS3) when the canopy was fully closed and dense (*f*≥0.9) corresponding to 35, 50, and 75 DAT.

**Fig. 2.**
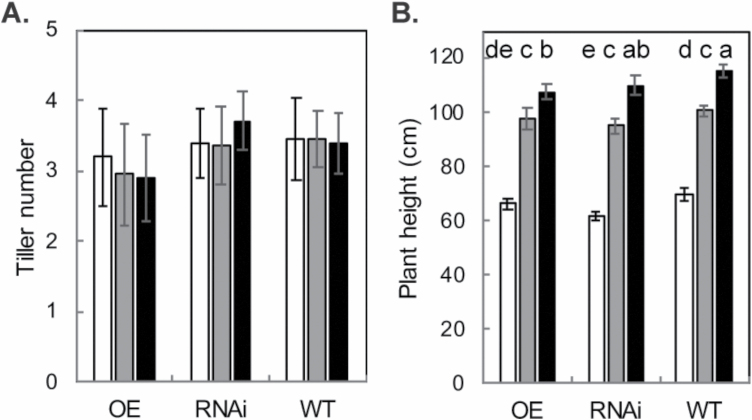
(A) Tiller number and (B) plant height recorded for rice overexpressing *psbS* (OE), down-regulating *psbS* (RNAi), and the wild type (WT) at the three growth stages, GS1 (white), GS2 (grey), and GS3 (black). Five plants per plot were sampled. Error bars denote the SEM (*n*=4) whilst letters indicate significant differences between genotype and growth stage according to ANOVA and Tukey’s multiple comparisons test (*P*<0.05).

Tiller number and plant height were recorded throughout development (Fig. 2). There were no significant differences in tiller number at any growth stage; however, there were significant differences in plant height between the genotypes and growth stages. The RNAi line had the lowest plant height compared with the OE and WT lines at GS1 and GS2. but intermediate at GS3, whereas the WT was the tallest at all growth stages. The differences in plant height did not correspond to any significant differences between the dry weights of the OE, RNAi, and WT lines at either growth stage. The dry weight of all the plants were significantly greater at GS3 compared with GS2 (*P*<0.001), but no differences were noted between genotypes at any growth stage (not shown).

**Fig. 3.**
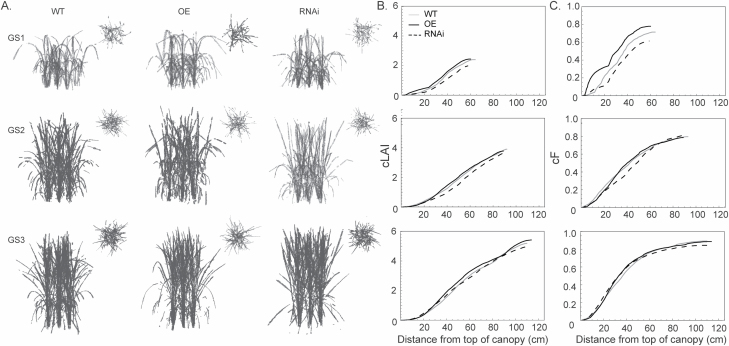
Physiological features in rice canopies with altered levels of PsbS. (A) The reconstructed canopies of WT, OE, and RNAi rice lines at GS1, GS2, and GS3. The main figure shows the side-on view, whilst the inset shows the top-down view. (B) The modelled cumulative leaf area index (cLAI); the area of leaf material (or mesh area) per unit ground area through the canopy. The steepness of the curve indicates how much leaf material is present at each layer. (C) The modelled cumulative fractional interception (cF); the distribution of light interception throughout the canopy. The steepness of the curve indicates a greater light interception at that canopy position.

To further assess structural properties, the canopies were reconstructed *in silico* (Fig. 3A). Visually, this indicates changes in leaf angle between the canopies, with more horizontal, curled leaves in the WT and OE lines, particularly at GS1, and a more upright leaf stature in the RNAi line throughout. To determine how this influences canopy function, cLAI can be determined as the amount of leaf area per unit ground area (calculated as mesh area) throughout the canopy depth (Fig. 3B). The steepness of the curves indicates the greater amount of leaf material present at a given canopy position. This indicates a larger amount of leaf material present at the top of the canopy at GS1 and the bottom of the canopy at GS3 in the overexpressor, corresponding to the presence of horizontal leaf material, or curled leaves. At GS2 and GS3, the top 20 cm of all three canopies indicates similar levels of leaf material present. Following this, there is an increase in leaf material in the OE line throughout the middle portion of the canopy at all three growth stages. Although visually different, cLAI profiles are similar between the WT and OE lines throughout development. Previously it has been shown that the accuracy of canopy reconstruction is sufficient to represent manual LAI measurements (Burgess *et al.*, 2015). This could not be performed during this experiment due to restrictions on transferring genetically modified material. Although there was no significance difference between tiller number and dry weight between the lines, the plant height and the total LAI (seen as the cLAI value at maximum depth) of the OE, RNAi, and WT lines showed that OE rice canopies accumulated greater leaf area at all growth stages (Fig. 3).

To see how changes in structural traits influence the absorption of light, cF was calculated (Fig. 3C). The steepness of the curve indicates a greater amount of light interception at a given canopy depth. At GS1, large differences are seen between the three lines, with the OE line achieving a much greater light interception, particularly pronounced within the top portion of the canopy. The WT shows a more even interception of light throughout canopy depth; whilst the RNAi line intercepts the least amount of light, particularly within the top portion of the canopy. At GS2, both the OE and WT lines show similar profiles of cF, whereas the RNAi line has a reduced light interception in mid canopy layers, and increased interception at the bottom of the canopy. By GS3, all lines show similar profiles of cF. It is also possible to calculate the average light intensity reaching leaf material of each of the canopies (Supplementary Fig. S2).

### *PsbS* does not alter steady-state photosynthesis but does increase NPQ capacity

**Fig. 4.**
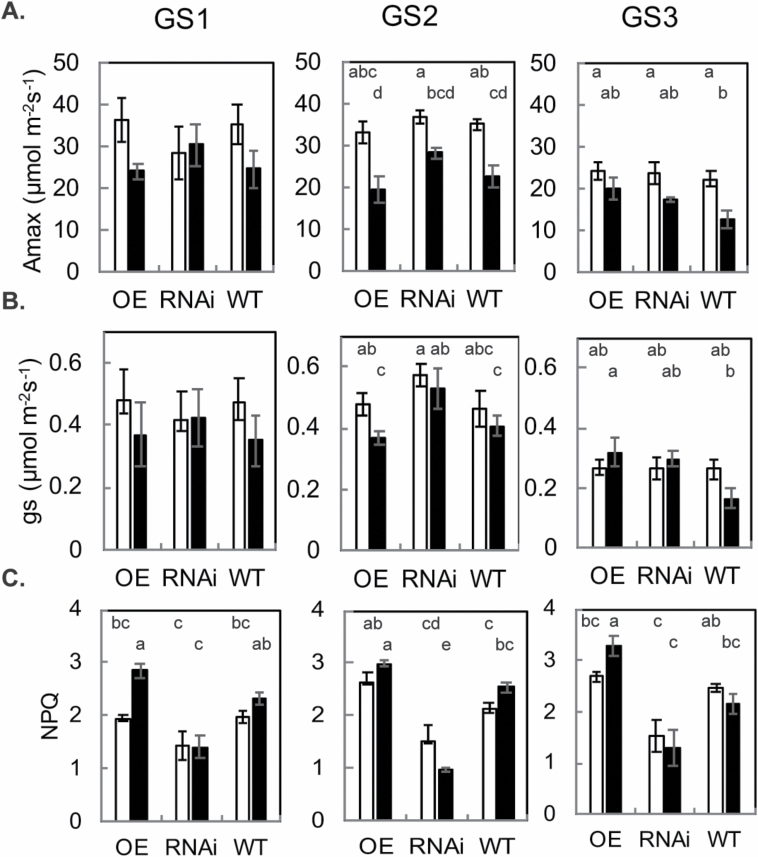
A selective comparison of (A) maximum carbon assimilation (*A*_max_), (B) stomatal conductance (*g*_s_), and (C) non-photochemical quenching (NPQ) measured at 2000 µmol m^–2^ s^–1^ at the top (white) and bottom (black) of the canopies of rice overexpressing *psbS* (OE), down-regulating *psbS* (RNAi), and the wild type (WT) at three growth stages, GS1, GS2, and GS3. Error bars denote the SEM (*n*=4) whilst letters indicate significant differences at each growth stage according to ANOVA and Tukey’s multiple comparisons test (*P*<0.05)

**Fig. 5.**
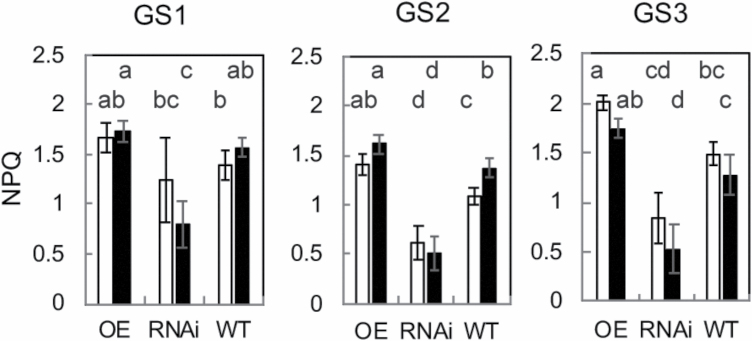
Non-photochemical quenching (NPQ) following 1 min of illumination at 1500 µmol m^–2^ s^–1^ at the top (white) and bottom (black) of the canopies of rice overexpressing *psbS* (OE), down-regulating *psbS* (RNAi), and the wild type (WT) at three growth stages, GS1, GS2, and GS3. Error bars denote the SEM (*n*=4) whilst letters indicate significant differences at each growth stage according to ANOVA and Tukey’s multiple comparisons test (*P*<0.05).

Overexpression or down-regulation of *psbS* resulted in differences in NPQ but had limited effects on steady-state photosynthesis and stomatal conductance (Fig. 4). There were no significant differences in terms of maximum carbon assimilation or stomatal conductance (*A*_max_ and *g*_s_ at 2000 µmol m^–2^ s^–1^) between the OE, RNAi, and WT lines at GS1. At both GS2 and GS3, the top canopy layer had higher maximum carbon assimilation compared with the lower canopy layer, as expected (e.g. Burgess *et al.*, 2015; Townsend *et al.*, 2018a). At all stages, NPQ was higher in the OE compared with the WT and RNAi lines, similar to results seen by Hubbart *et al*. (2012, 2018). To explore how PsbS affects the speed of NPQ formation, NPQ induction was performed by exposing dark-adapted leaves to 1500 µmol m^–2^ s^–1^. The NPQ following the first minute of illumination can be used as a proxy for formation rate (Fig. 5). This indicates that the overexpression of *psbS* significantly increased the rate of formation whilst down-regulation significantly reduces the rate of formation, relative to the WT, which is consistent across all growth stages.

### Photoprotective NPQ is greater in the lower regions of the canopy

Plants were considered photoinactivated when qP_d_ was <0.98. All light tolerance curves are given in Supplementary Fig. S3. Supplementary Fig. S4 indicates the light intensity that caused photoinactivation in 50% of the leaves within the canopy layer (*I*_50%_), which was derived from the light tolerance curves, and thus can provide an indication of phototolerance. At all growth stages there was a tendency for the OE line to have an increased *I*_50%_ relative to the other lines with the exception of GS3 where the canopy had a lower cLAI (Fig. 3).

**Fig. 6.**
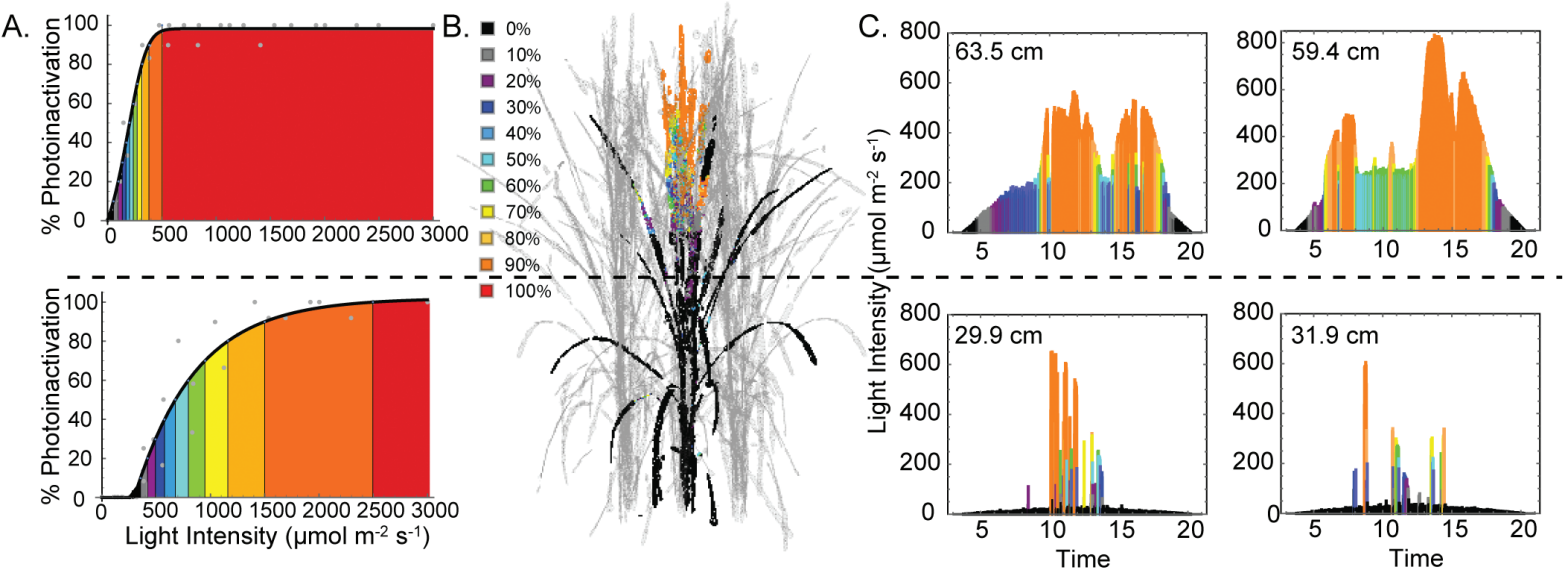
Light tolerance curves combined with canopy light profiles enable the visualization of photoinactivation according to canopy position. (A) Wild-type (WT) rice light tolerance curves for the canopy top and bottom calculated using the fluorescence routine on the Junior-PAM (Heinz Walz). Plants were considered photoinactivated when qP_d_ was <0.98. The light intensity ranges that correspond to a set photoinactivation per canopy top or bottom are colour coded in 10% bins. (B) A representative reconstructed WT rice canopy at GS3 with a single plant in bold, with colour corresponding to photoinactivation of leaf material dependent upon light intensity calculated from the inverse of light tolerance curves (A) using Equation 3 (Materials and methods). (C) The light intensity during the course of a day at four representative canopy positions, with the height of each canopy location from the ground given in the top left corner of each graph, calculated using ray-tracing techniques. Light signatures are colour coded using the corresponding intensity values (A and B).

The phototolerance curves in themselves do not give a full understanding of the role of PsbS in protective NPQ as they do not account for altered canopy structure and the resulting changes in the light environment. This can be achieved by modelling approaches that take into account high resolution changes in light intensity resulting from unique differences in structural traits. Figure 6 represents how the light tolerance curves can be integrated with canopy reconstructions in order to determine the role of PsbS in pNPQ at the canopy scale. The light tolerance curves can be used to determine the corresponding light intensities at which a given proportion of leaf material can be considered to be photoinactivated by separating the curves into 10% photoinactivation bins (i.e. *I*_10–100%_; denoted by different colours in Fig. 6A). In combination with ray tracing, this can visualized throughout the canopy structure to indicate regions of the canopy where photoinactivation is greater by colour-coding leaf material according to the corresponding light intensity values (Fig. 6B). Alternatively, given profiles of light throughout the day at different canopy positions, the period of time that each specific location spends under a given amount of photoinactivation can be visualized (e.g. Fig. 6C for four randomly selected canopy positions).

**Fig. 7.**
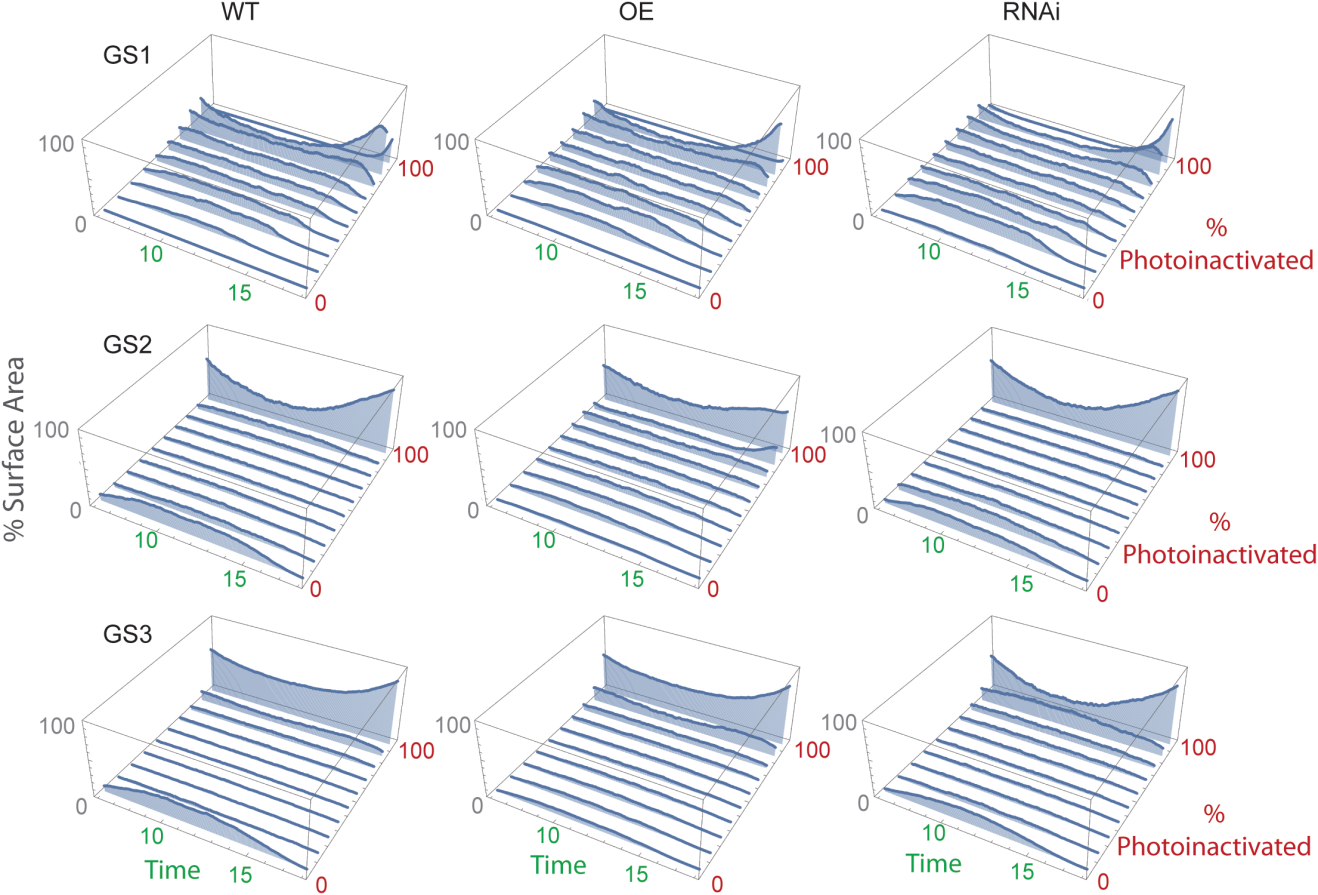
Profiles of photoinactivation (expressed in terms of the percentage of leaves photoinactivated) according to total surface area and time of day between 06.00 h and 18.00 h for rice overexpressing *psbS* (OE), down-regulating *psbS* (RNAi), and the wild type (WT) at three growth stages, GS1, GS2, and GS3.

Taken together, this allows the portion of the canopy (calculated as a percentage of the total surface area) under different levels of photoinactivation to be calculated (Fig. 7). At GS1, when the canopy was just starting to develop, most of the leaves were experiencing some sort of photoinactivation. This appears to be more pronounced in the RNAi line, seen as an increased percentage of surface area within the *I*_90%_ bin. As expected, photoinactivation is more pronounced at midday and reduced at sunrise and sunset for all lines and growth stages as a result of diurnal solar movement. At all growth stages, but particularly visible at GS2 and GS3, the OE line shows a reduced percentage of surface area within the highest photoinactivation bin (i.e. *I*_100%_), followed by the WT then RNAi. This is partly explained by the higher average light intensity reaching leaf material in the RNAi line relative to the other lines (see Supplementary Fig. S2 for average light intensities at 12.00 h). The latter growth stages show a reduced amount of leaf material affected by photoinactivation in all lines, as expected due to canopy closure and self-shading.

## Discussion

NPQ can have both beneficial and negative effects on canopy productivity. Whilst photoprotection is required to alleviate ‘pressure’ on the photosynthetic membrane and prevent damage, overprotection may also reduce photosynthetic efficiency due to the lag between changes in light intensity and response. The pNPQ protocol allows the non-invasive assessment of the protective capacity of NPQ and, when combined with high-resolution reconstruction and modelling, enables, for the first time, the whole canopy pNPQ to be assessed (Ruban, 2017). It not only allows the characterization of light tolerance curves which can indicate the early onset of photoinactivation (Supplementary Fig S3), but also enables the level of photoinactivation to be calculated simultaneously at all positions within a canopy throughout the day, whilst accounting for changes in structural characteristics (Figs 6, 7). The approach we have taken here is especially relevant to the spatiotemporally complex light environments within plant canopies where the behaviour and effect of dynamic processes such as NPQ have been difficult to predict.

In this study, pNPQ was analysed in rice plants with either up-regulated, WT, or down-regulated *psbS*. The rice *psbS* OE lines have been shown to have an increased weight and grain yield in fluctuating light, whilst the RNAi lines are usually smaller (Hubbart *et al.*, 2012). Here, we have advanced on these previous studies by using high-resolution canopy reconstruction combined with NPQ measurements.

The power of this approach is seen in Figs 6 and 7 which demonstrate that, contrary to expectations, protective quenching (pNPQ) was higher in the lower parts of the canopy regardless of the genotype. This is perhaps unexpected because leaves lower in the canopy are typically shade or low light acclimated, which means they typically have a lower photosynthetic capacity and are less geared toward processing high light levels, resulting in less need for a high photoprotective capacity. On the other hand, leaves in the upper portions of the canopy should be able to tolerate high light levels without the risk of photoinactivation (though they are more photoinactivated, Fig. 6). This is seen in the accumulation of xanthophyll cycle pigmentation at high and low growth light intensities. The explanations for this observation may be critical when discussing the trade-offs between using light efficiently for photosynthesis and preventing photoinactivation and photooxidation (discussed at length in Kromdijk *et al.*, 2016; Hubbart *et al.*, 2018; Murchie and Ruban, 2020; Wang *et al.*, 2020). First the lower leaves should exploit brief periods of high light known as sunflecks (Fig. 6C) which are more common in lower regions. To do this, they need to rapidly move from a low photosynthetic rate to a high one, and, in order to do this without damaging PSII, greater photoprotective quenching would be needed. It has been accepted for many decades that under low light (and low CO_2_ assimilation rates), high levels of photoinactivation and photodamage are costly for the leaf carbon budget since repair to the D1 protein within the RC requires energy investment. Therefore, this may be a beneficial adaptation to protect PSII under low light. The higher inactivation at high light may represent the increased probability of potential damage due to higher photon dose but this does not explain the lower levels of light tolerance.

The reconstruction method has shown greater accumulation of leaf material in the OE line, particularly within mid-canopy layers, and a more sparse, upright leaf stature in the RNAi line in upper canopy layers (Fig. 3). Manual measurements such as tiller counts, plant height, and above-ground dry weights were insufficient to detect these differences. This is not unexpected as, despite similar heights, dry weight, and total LAI of all three lines, the arrangement of leaf material throughout the canopy depth was altered, which cannot be detected using traditional manual measurements. This is important as the specific structural properties of the canopy are critical in determining the absorption of light and the resulting load on the photosynthetic membrane (Burgess *et al*., 2015, 2017*a*; Townsend *et al.*, 2018a). This is reflected in the profiles of cumulative fractional interception (cF), whereby more light is intercepted in upper canopy positions of the WT and the OE line compared with the RNAi line, particularly at GS1 and GS2, despite similar total fractional interception values when measured manually with a ceptometer (Fig. 3C; Supplementary Fig. S1).

The pNPQ protocol in itself is not sufficient to characterize the role of PsbS in rice canopy NPQ. Whilst previous studies have shown the increased protective capacity of NPQ attributed to increased levels of *PsbS* in *A. thaliana* (Ware *et al.*, 2014), the same relationship between PsbS overexpression or down-regulation and phototolerance, seen as the *I*_50%_ value, was not replicated here (Supplementary Fig. S4). This is likely to be due to the differences in structural properties of the Arabidopsis rosette versus the complex, 3D rice canopy. Structural differences contribute to an increased average light intensity in the RNAi line, relative to the other lines, due to a more upright, sparse canopy (Fig. 3). This may in part explain the higher than expected *I*_50%_ values of the RNAi line relative to the OE line, particularly in the top layer of the canopy at GS1. This could be attributed to acclimation of leaf material to higher irradiance levels (Retkute *et al.*, 2015; Townsend *et al.*, 2018a). However, despite this, the more open structure of the RNAi lines is also likely to have contributed to the more extreme photoinactivation profiles (Fig. 7). Similarly, the more enclosed, dense canopy of the OE line at all developmental stages will have contributed to the less extreme photoinactivation profiles, as a result of early canopy growth and self-shading. This is consistent with previous findings in the rice OE lines (Hubbart *et al*., 2012, 2018). Together, this indicates the importance of accounting for realistic canopy structures when scaling up leaf-level processes, as measurement of the process in itself is not always sufficient to characterize canopy function. Offsetting between canopy architecture and NPQ characteristics is a possible outcome.

It was previously reported that there is an ontological effect of pNPQ capacity, whereby older Arabidopsis leaves can tolerate higher levels of light (Carvalho *et al.*, 2015). Similar results have been found here, whereby the bottom layers of all three rice canopies at GS2 and GS3 showed higher light tolerance than the top layers (Supplementary Fig. S4). However, within rice plants, leaves grow from the base and extend up, and thus the same leaf can sit within the top half and bottom half of the canopy. To account for this, careful random selection of leaves during measurements took place. However, for a true assessment of pNPQ capacity of canopies, both the ontological effect and spatial differences in pNPQ capacity at the individual leaf level should be taken into account.

This and previous studies indicate a potential 2-fold advantage of manipulating NPQ in order to improve photosynthetic efficiency and yield production (Hubbart *et al*., 2012, 2018; Kromdijk *et al.*, 2016). First, overexpression of genes central to the regulation on NPQ are able to increase the phototolerance of leaf tissue and increase the speed of formation and relaxation of NPQ to reduce the lag time between changes in light intensity and response, thus conferring cellular level improvements. Secondly, manipulation of NPQ could lead to indirect changes in canopy structural properties which could affect both the quantity and the arrangement of leaf material and provide an advantage in terms of early canopy expansion (Fig. 3; Hubbart *et al*., 2012, 2018).

The complexity of the field environment means that the actual influence of NPQ on canopy function (e.g. in terms of canopy carbon gain in a variety of environments) is yet to be fully assessed. The light intensity within crop canopies has high spatiotemporal variability and is dependent upon features such as organ dimensions, angles, and the quantity of leaf material present. Many studies do not account for the heterogeneity in canopy structure, which is the first stage towards analysing the response to fluctuating light (Bielczynski *et al.*, 2017; Burgess *et al.*, 2017*b*). Here, this has been overcome through the use of high-resolution reconstructions which can accurately capture small variations in structural traits. Confounding this further are environmental factors including solar movement, the presence of cloud cover, and wind which can induce conformational changes to structural properties, further altering the light environment in the canopy (Burgess *et al.*, 2019). Whilst a full characterization of the rapidity and magnitude of changes in light intensity is not known, it is likely that a rapid biochemical response, such as NPQ induction or relaxation, will be critical in preserving and maximizing canopy function.

In conclusion, the overexpression of *psbS* was associated with increased capacity for NPQ. However, all genotypes indicate that lower canopy layers have a higher phototolerance, regardless of their inherent capacity for NPQ. This indicates a mechanism geared towards increased protection for leaves acclimated to low light and experiencing low levels of light interspersed with high peaks of intensity.

## Supplementary data

The following supplementary data are available at *JXB* online.

Fig. S1. Fractional interception.

Fig. S2. Frequency of light intensity according to the fraction of surface received at 12.00 h in the canopy top and bottom for rice plants overexpressing *psbS* (OE), down-regulating *psbS* (RNAi), and the WT at three growth stages.

Fig. S3. Relationship between the percentage of photoinactivated leaves and light intensity for rice plants overexpressing *psbS* (OE), down-regulating *psbS* (RNAi), and the WT at three growth stages.

Fig. S4. A comparison of the light intensities at which 50% of the leaves are photoinactivated (*I*_50%_) at the top (white) and bottom (black) layers of the canopy of rice overexpressing *psbS* (OE), down-regulating *psbS* (RNAi), and the WT at three growth stages.

eraa411_suppl_Supplementary_File

## Data Availability

Authors will make all data available on request.

## Abbreviations

AL: actinic light
cF: cumulative fractional interception
cLAI: cumulative leaf area index
FR: far red
OE: overexpressor
pNPQ: protective non-photochemical quenching
PsbS: PSII subunit S
qE: energy-dependent quenching
qP: quantum coefficient of photochemical quenching in the light
qP_d_: quantum coefficient of photochemical quenching in the dark following light exposure
RC: reaction centre
SP: saturating pulse
WT: wild type.
