# Supplementary material for: Photoprotective energy dissipation is greater in the lower, not the upper, regions of a rice canopy: a 3D analysis
Source: J Exp Bot. 2020 Sep 9;71(22):7382–92. doi: 10.1093/jxb/eraa411 (PMC7906788; doi:10.1093/jxb/eraa411)
Supplement: eraa411_suppl_Supplementary_File [file eraa411_suppl_supplementary_file.pdf]

Supplementary Material

Supplementary Figure S1

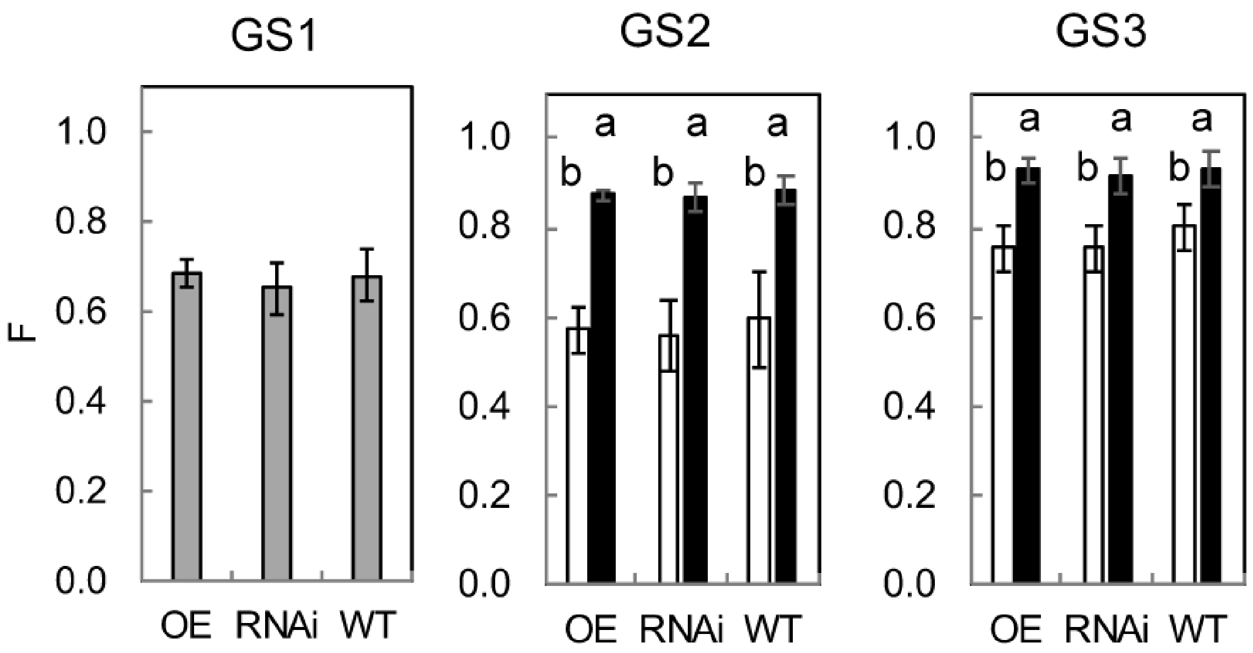

Supplementary Figure S1. Fractional interception, F was measured using an AccuPAR LP-80 ceptometer (Decagon Devices, Washington, USA) within the plots of rice overexpressing *PsbS* (OE), downregulating *PsbS* (RNAi) and wildtype (WT) at three growth stages (GS) at GS1, GS2 and GS3. At GS1, F was measured for the whole canopy as one (grey). At GS2 and GS3, F was measured at the top layer (white) and for the total canopy (black) of the canopies. Error bars denote the standard error of the mean (n=4) whilst letters indicate significant differences at each growth stage according to ANOVA and Tukey's multiple comparisons test (P<0.05).

Supplementary Figure S2

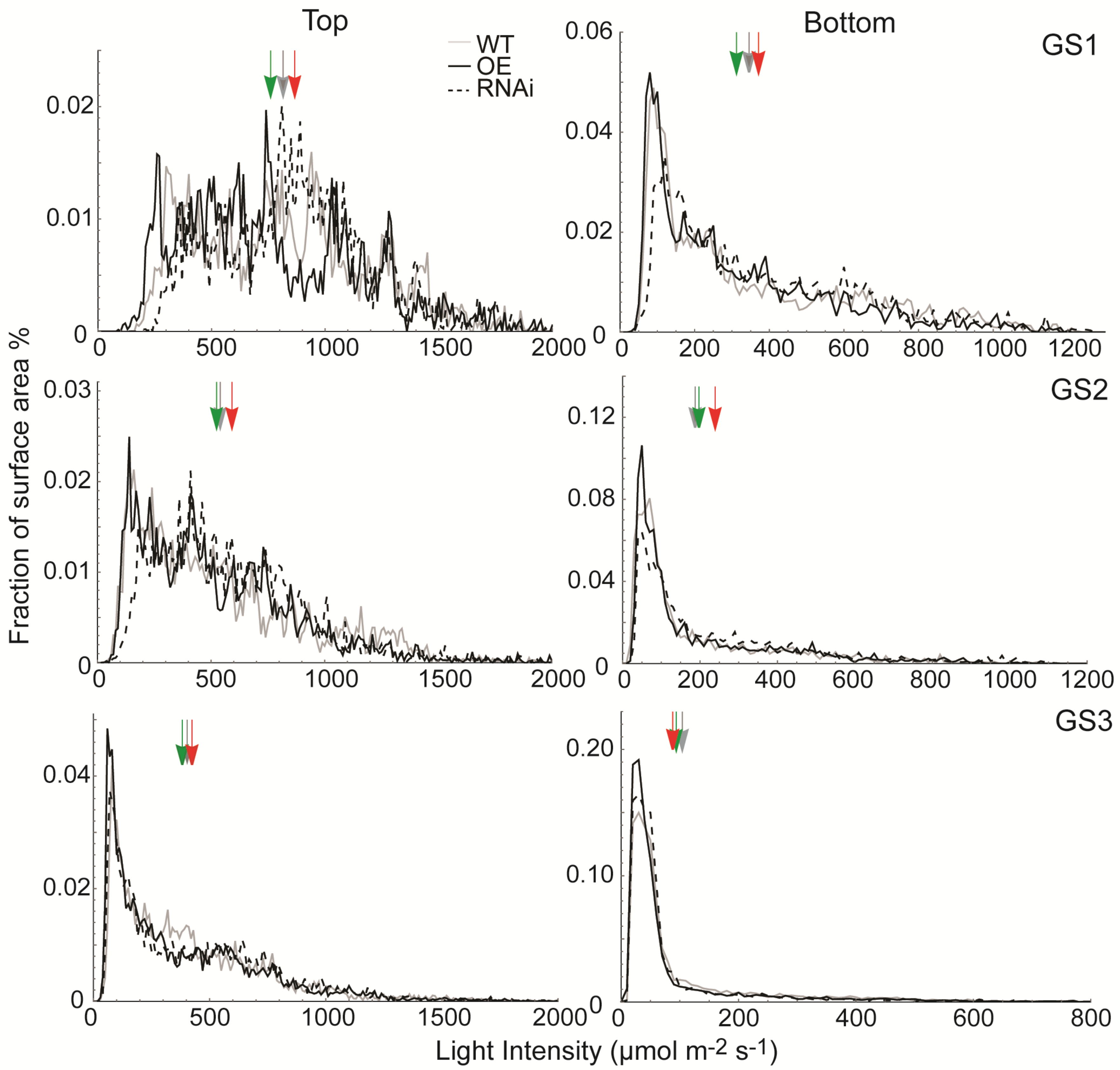

Supplementary Figure S2. Frequency of light intensity according to the fraction of surface received at 1200h in the canopy top and bottom for rice plants overexpressing *PsbS* (OE); downregulating *PsbS* (RNAi) and the WT at three growth stages. The grey, green and red arrows indicate the average light intensity at each canopy layer for WT, OE and RNAi canopies, respectively.

Supplementary Figure S3

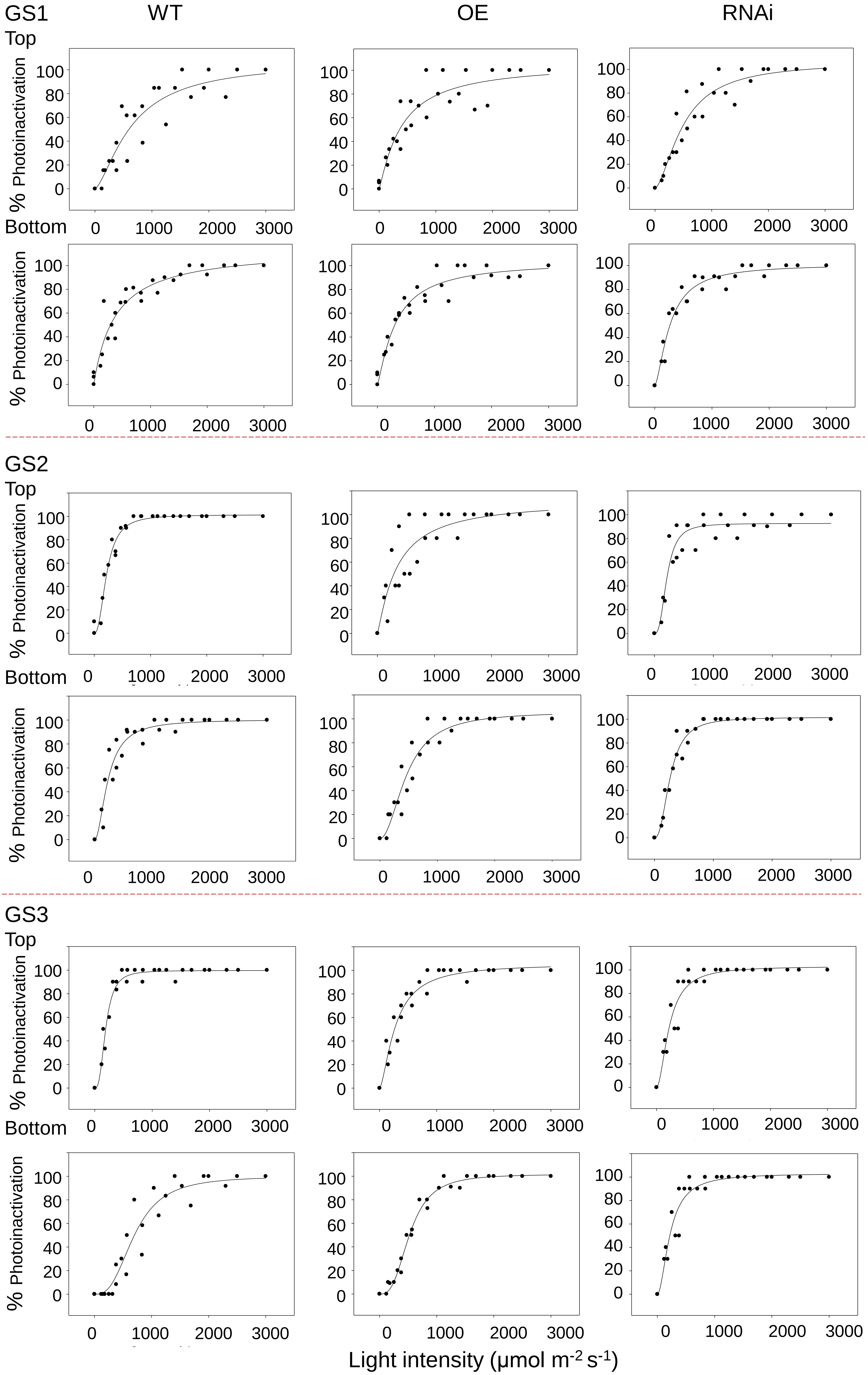

Supplementary Figure S3: Relationship between the percentage of photoinactivated leaves and light intensity for rice plants overexpressing *PsbS* (OE); downregulating *PsbS* (RNAi) and wildtype (WT) at the three growth stages (GS), GS1, GS2 and GS3. Data was fitted using a sigmoidal hill function in Mathematica (Version 10.0; Wolfram, Oxford, UK).

Supplementary Figure S4

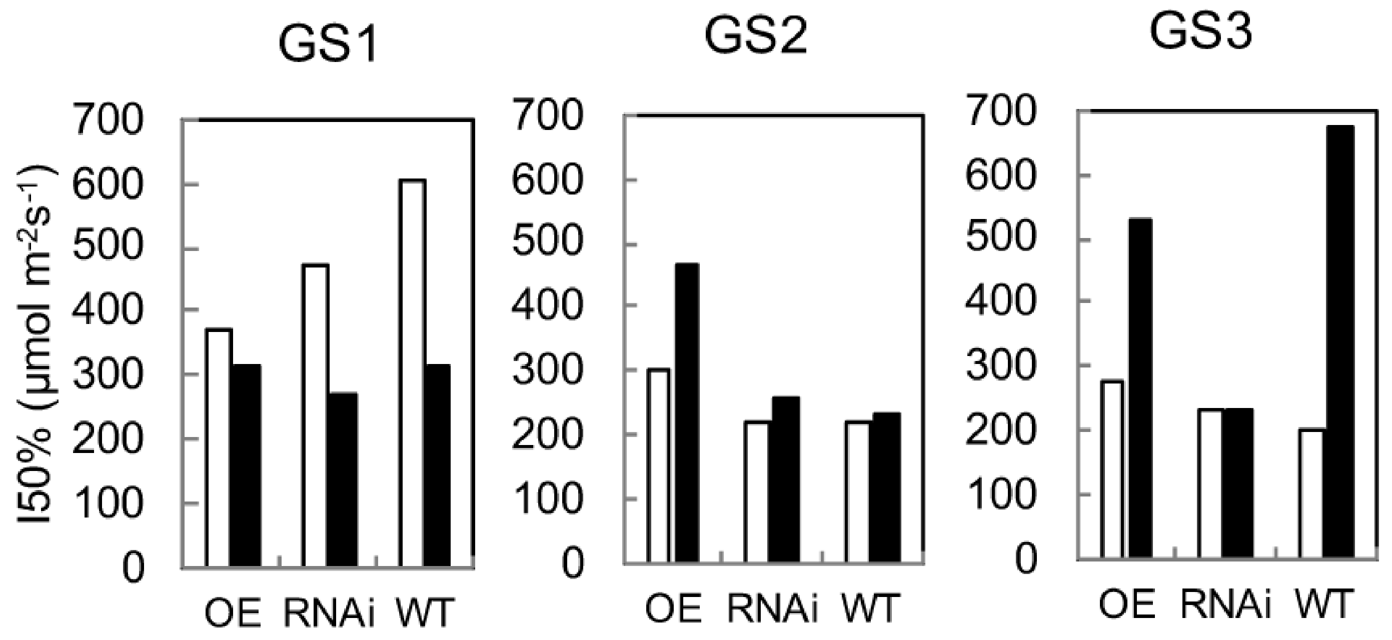

Supplementary Figure S4. A comparison of the light intensities at which 50% of the leaves are photoinactivated (I50%) at the top (white) and bottom (black) layers of the canopy of rice overexpressing *PsbS* (OE); downregulating *PsbS* (RNAi) and wildtype (WT) at the three growth stages (GS), GS1, GS2 and GS3.
